# Genetic engineering to improve resistance against heavy metal stress in *Synechocystis* sp. PCC 6803

**DOI:** 10.1128/aem.02473-25

**Published:** 2026-01-21

**Authors:** Yantao Yang, Chen Zheng, Lin Gao, Xinyan Jiang, Jingling Xu, Wenjing Zhang, Ying Peng, Jiaxin Han, Menghao Sun, Wenxian Ma, Wei Shi, Xihui Shen

**Affiliations:** 1 State Key Laboratory for Crop Stress Resistance and High-Efficiency Production, Shaanxi Key Laboratory of Agricultural and Environmental Microbiology, College of Life Sciences, Northwest A&F University 213667, Yangling, China; Colorado School of Mines, Golden, Colorado, USA

**Keywords:** *Synechocystis *sp. PCC 6803, genetic engineering, functional elements, heavy metal stress, stress resistance

## Abstract

As urbanization accelerates, the severity of heavy metal pollution in soil and water bodies intensifies. Cyanobacteria possess significant water remediation capabilities. To promote their application in environmental bioremediation, the selection and genetic modification of superior cyanobacterial strains have become increasingly important. In this study, we successfully constructed transgenic *Synechocystis* sp. PCC 6803 strains expressing exogenous *mntH* (manganese transporter protein), *HMP3* (human metallothionein), and *sodA* and *sodC* (superoxide dismutases) genes, respectively. We systematically evaluated the tolerance of these transgenic strains against stress induced by Cd^2+^, Pb^2+^, and Cr^6+^ heavy metal ions. Under normal conditions, no significant differences in growth were observed between wild-type and transgenic *Synechocystis* sp. PCC 6803 strains. When exposed to heavy metal stress, the growth of all strains was severely inhibited. However, compared to the wild type, the transgenic strains exhibited significantly improved growth performance, accompanied by marked increases in intracellular chlorophyll, carotenoids, phycobiliprotein, and total protein contents. Further analysis revealed that this improvement correlated with significantly enhanced activities of superoxide dismutase (SOD) and catalase (CAT), which effectively mitigated reactive oxygen species (ROS) levels generated by heavy metal ions, thereby enhancing the tolerance of the transgenic strains to heavy metal stress. By introducing effective exogenous genes, our study successfully yielded heavy metal-tolerant strains of *Synechocystis* sp. PCC 6803. This not only validated the efficacy of the four exogenous functional genes in enhancing cyanobacterial stress resistance but also provided theoretical and technical support for developing more resilient cyanobacterial chassis cells or applying them to bioremediation.

**IMPORTANCE:**

Cyanobacteria, which possess photoautotrophic capacity, demonstrate excellent capabilities in water remediation. As ideal species integrating both photosynthetic carbon fixation and bioremediation functions, cyanobacteria have garnered significant attention for environmental bioremediation applications. Consequently, the selection of superior cyanobacterial strains and genetic engineering for improvement have become increasingly critical to advance practical application in ecological remediation processes. *Synechocystis* sp. PCC 6803 possesses a well-defined genetic background and a natural DNA transformation system, making it an ideal platform for gene editing and metabolic engineering. In this study, we successfully constructed transgenic *Synechocystis* sp. PCC 6803 strains expressing exogenous genes encoding MntH, HMP3, SodA, and SodC, respectively. The heavy metal resistance of transgenic strains was significantly improved. This study underscores the efficacy of exogenous functional genes in improving cyanobacterial stress resistance and offers both theoretical and technical foundations for the development of more robust cyanobacterial chassis cells.

## INTRODUCTION

With the acceleration of industrialization and urbanization process, heavy metal contamination in aquatic environments has emerged as a global challenge threatening ecological security and human health. The persistent accumulation of heavy metal ions, such as lead (Pb), cadmium (Cd), and chromium (Cr), in aquatic environments not only disrupts the equilibrium of aquatic ecosystems but also poses risks to human health, including neurotoxicity and carcinogenicity through bioaccumulation in the food chain (1). Conventional heavy metal remediation techniques, including chemical precipitation and ion exchange methods, are limited by their high costs and potential for secondary pollution (2). Consequently, bioremediation—a green, efficient, economical, and highly promising approach—has emerged as a crucial research direction for pollution remediation (3, 4).

Recent studies have revealed that cyanobacteria possess a remarkable capacity for heavy metal adsorption, attributable to their distinctive cell wall structure and metabolic properties. Their cell walls are abundant in negatively charged functional groups, including hydroxyl and carboxyl groups, which facilitate the effective accumulation of heavy metal ions, such as Pb^2+^ and Cd^2+^, through ion exchange and complexation (4, 5). These biosorbents not only immobilize heavy metals directly but also stably sequester pollutants intracellularly via metabolic pathways. Furthermore, their composite formulations can simultaneously achieve heavy metal removal and algal bloom control (6, 7). Compared to conventional methods, cyanobacterial remediation technology presents notable advantages, including cost-effectiveness and environmental friendliness (8). For instance, studies have shown that *Anabaena subcylindrical* and *Nostoc* sp. can effectively remove harmful metals, such as Pb^2+^, Co^2+^, Mn^2+^, and Fe^2+^, from wastewater (9, 10). EI Hameed et al. reported that *Nostoc muscorum* could thrive in wastewater containing 0.5 mg/L Cd^2+^ and achieve a Cd^2+^ removal efficiency of up to 93.4% within 21 days (11). Franco et al. demonstrated that *Nostoc paludosum* could remove 73–96% of inorganic Hg^2+^ and 73–95% of organic mercury from wastewater within 30 days (12), offering a sustainable approach to managing heavy metal pollution.

Research has shown that the model cyanobacterium *Synechocystis* sp. PCC 6803 efficiently accumulates heavy metals through multiple mechanisms, including ion exchange, surface complexation, and biomineralization, mediated by components such as cell wall polysaccharides, extracellular polymeric substances (EPS), and metal transporters (13). Its photosynthetic autotrophic nature further enables synergistic heavy metal fixation and resource recovery, offering a sustainable approach to environmental remediation (14). As the first photosynthetic model organism with complete genome sequencing (15), PCC 6803 possesses a well-defined genetic background and a natural DNA transformation system, making it an ideal platform for gene editing and metabolic engineering (16, 17). Studies have shown that introducing exogenous genes via homologous recombination or CRISPR-Cas9 technology can significantly enhance its stress resistance (18). Despite the existence of several patents for utilizing cyanobacteria as platforms for large-scale metabolite production, the development of synthetic biology tools for cyanobacteria currently lags behind those available for heterotrophic model strains (19). Therefore, it is crucial to screen high-performance chassis cells, expand the toolkits, and optimize libraries of transferable functional elements to advance the application of *Synechocystis* in bioremediation and the industrialization of photosynthetic carbon fixation technologies.

With the advancement of research on biological environmental adaptation mechanisms, an increasing number of functional proteins have been identified. For instance, the manganese transporter MntH, as a bacterial homolog of the NRAMP family (natural resistance-associated macrophage protein) (20), plays a central role in pathogen stress resistance. For example, *Salmonella* regulates intracellular manganese concentration via MntH to overcome the host nutritional immunity (21). Superoxide dismutase (SOD), an antioxidant metalloenzyme present in living organisms, decomposes ROS to mitigate oxidative damage, thereby protecting cellular components (22). Metallothioneins (MT) are low-molecular-weight cysteine-rich metal-binding proteins widely distributed in living organisms (23). The expression of MT and its oligomer in *Escherichia coli* confirmed that they significantly enhanced Cd^2+^ and As^3+^ accumulation capacity, effectively boosting bioremediation efficiency (24). These studies on environmental adaptation-related functional proteins provide valuable materials and methodological references for cyanobacterial improvement, offering potential as transferable functional elements for engineering stress-resistant cyanobacteria.

This study employed the application-promising *Synechocystis* sp. PCC 6803 as a platform to investigate the expression of heterologous stress-resistant functional proteins, including MntH, MT, and SOD. The tolerance of transgenic cyanobacteria strains to heavy metal ion stresses, such as Cr^6+^, Pb^2+^, and Cd^2+^, was measured. This research provides a material and methodological basis for identifying transferable functional elements in cyanobacteria and for targeted cyanobacterial engineering.

## RESULTS

### Construction of transgenic *Synechocystis* sp. PCC 6803 strains

**Fig 1:**
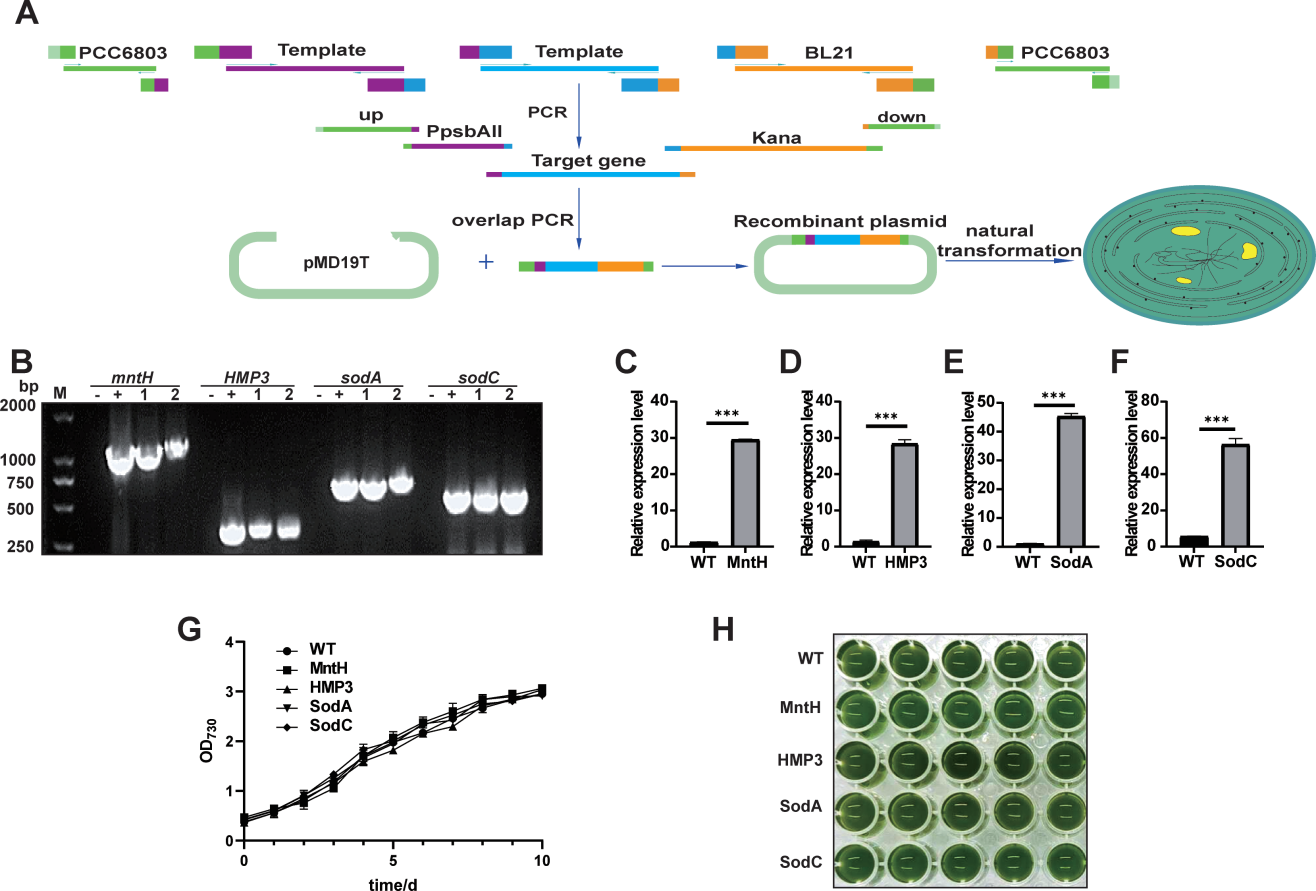
Construction of transgenic algal strains. (**A**) Schematic flowchart of transgenic algal strain construction. (**B**) PCR detection and identification of transgenic algal strains; M denotes marker. (**C–F**) qRT-PCR detection of *mntH*, *HMP3*, *sodA*, and *sodC* gene expression in transgenic algal strains, respectively. (**G**) Growth curves of transgenic algal strains in BG11 medium. (**H**) Growth status of cyanobacteria cultured for 10 days in BG11 medium. Data are shown as mean ± SEM (*n* = 3). ***P < 0.001.

The transgenic *Synechocystis* sp. PCC 6803 strains expressing genes of MntH from *Salmonella* typhimurium SL1344 (21), human metallothionein 3 (HMP3, GenBank: KJ897206.1) (25), and SodA and SodC from *E. coli* (26) were constructed as described in the “Materials and methods” section (Fig. 1A and B). The positive strains were subcultured until the genome was stable to obtain transgenic strains expressing MntH, HMP3, SodA, and SodC. qRT-PCR analysis confirmed the transcriptional expression of exogenous gene *mntH*, *HMP3*, *sodA*, and *sodC* in the corresponding transgenic strains (Fig. 1C and F). In normal BG11 medium, there was no significant difference between the growth of each transgenic strain and that of wild type (Fig. 1G and H), indicating that the expression of the four exogenous genes had no significant impact on the normal growth of *Synechocystis* sp. PCC 6803.

### Enhanced Cd^2+^ stress tolerance in transgenic *Synechocystis* sp. PCC 6803

**Fig 2:**
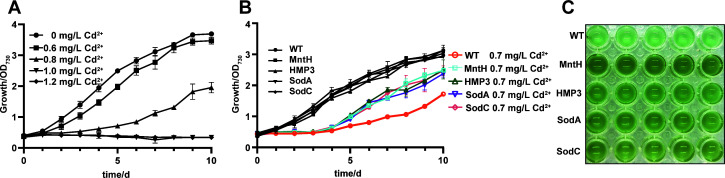
Effects of Cd^2+^ stress on the growth of wild-type and transgenic PCC6803 strains. (**A**) The wild-type PCC6803 was cultivated in BG11 medium with different concentrations of Cd^2+^. (**B**) Effects of 0.7 mg/L Cd^2+^ stress treatment on the growth of wild-type and transgenic strains. (**C**) Growth status of algal strains after 10 days of 0.7 mg/L Cd^2+^ stress treatment.

*Synechocystis* sp. PCC 6803 was cultured in BG11 medium with different concentrations of Cd^2+^. As expected, the growth of PCC 6803 was inhibited by Cd^2+^ (Fig. 2A), and higher concentration caused more serious inhibition and even cell death (Cd^2+^ > 1.0 mg/L). Based on this, the median lethal concentration (LC_50_) of Cd^2+^ to PCC 6803 was determined to be 0.6–0.8 mg/L. Therefore, 0.7 mg/L was selected as the treatment concentration for studying stress tolerance of Cd^2+^ in this study. As shown in Fig. 2B and C, under the stress of 0.7 mg/L Cd^2+^, the wild-type strain of PCC 6803 exhibits a serious growth inhibition, while the MntH, HMP3, SodA, and SodC transgenic strains show obvious growth advantages over the wild type. After 10 days of treatment, the inhibition rate of the wild-type strain reached 44.87%, while those of MntH, HMP3, SodA, and SodC transgenic strains were similar, all below 19% (Fig. S1A). The HMP3 strain exhibited a relatively lower inhibition rate, indicating that it possessed the greatest resistance to Cd^2+^ among these transgenic strains.

**Fig 3:**
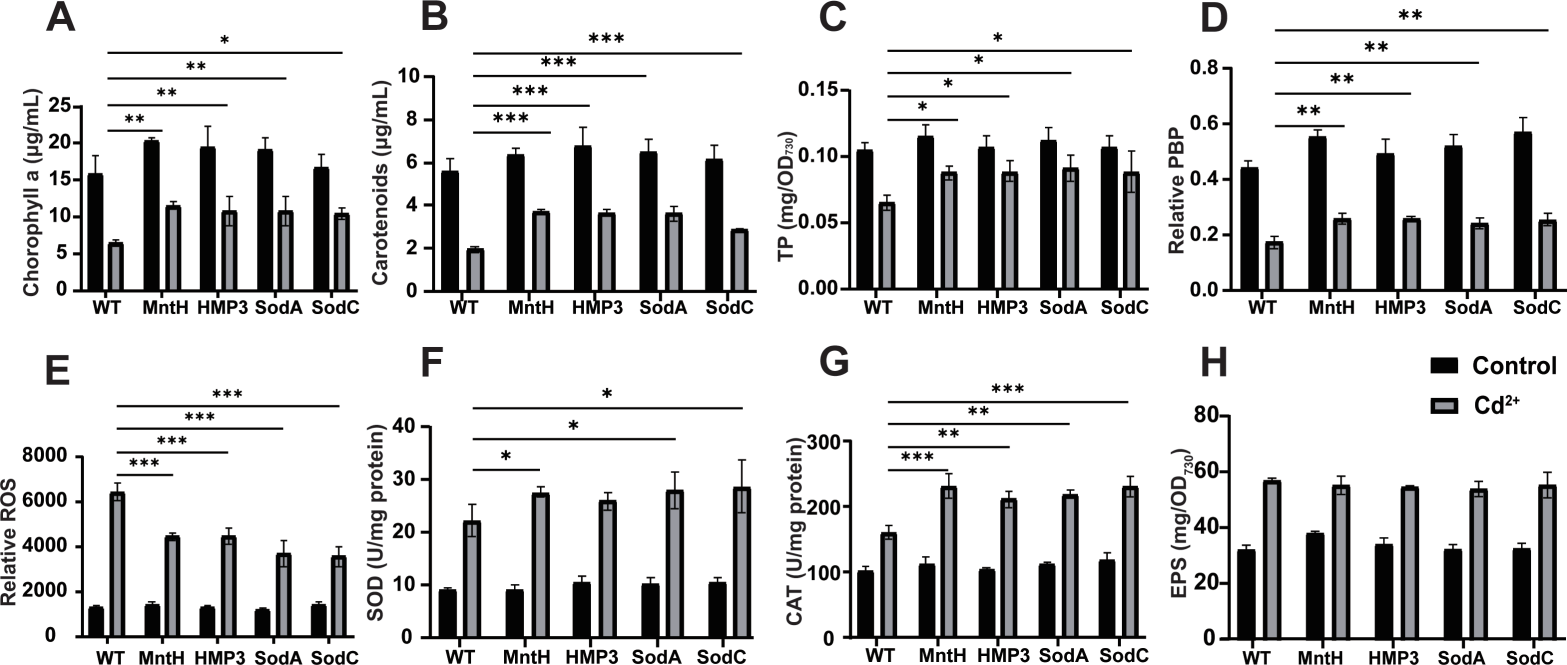
Effect of exogenous gene expression on the tolerance of PCC6803 to Cd^2+^ stress. After 10 days of treatment with 0.7 mg/L Cd^2+^, the chlorophyll (**A**), carotenoids (**B**), total protein content (**C**), relative PBP content (**D**), ROS level (**E**), SOD activity (**F**), CAT activity (**G**), and EPS content (**H**) of strains were measured. Data are shown as mean ± SEM (*n* = 3). *P < 0.05; **P < 0.01; ***P < 0.001.

We investigated the responses of transgenic and wild-type strains to Cd^2+^ stress by measuring relevant physiological indexes in cells after 10 days of treatment with 0.7 mg/L Cd^2+^. The damage rates of chlorophyll and carotenoids in the wild-type PCC 6803 reached 58.86% and 64.64%, respectively (Fig. 3A and B), after treatment. This may be attributed to the fact that Cd^2+^ promotes the degradation of photosynthetic pigments through ROS generation or enhanced activity of photosynthetic pigment enzymes, thereby disrupting the photosynthetic pigments in microalgae and affecting its growth (27). While the damage rates of chlorophyll in MntH, HMP3, SodA, and SodC transgenic strains were 43.48%, 44.55%, 43.8%, and 37.37%, respectively (Fig. 3A), those of carotenoids were 41.5%, 45.78%, 43.73%, and 53.17%, respectively (Fig. 3B), which were significantly lower than those in the wild type, indicating that these transgenic strains significantly reduced pigment damage under Cd^2+^ stress (Fig. 3A and B). Additionally, the total protein and phycobiliprotein (PBP) contents of PCC 6803 decreased significantly after Cd^2+^ exposure, and the damage rates reached 38.18% and 60.68%, respectively. However, the effects on total protein and PBP contents of four transgenic strains were relatively small (Fig. 3C and D). It is speculated that exogenous metal-binding proteins or SOD in the transgenic strains may alleviate Cd^2+^ toxicity by binding and immobilizing heavy metal ions or by enhancing antioxidant enzyme activities, thereby mitigating the effects of Cd^2+^ on cellular pigments and protein expression.

To further clarify the mechanisms underlying Cd^2+^ tolerance differences among algal strains, we measured ROS levels in each strain after Cd^2+^ stress. After treatment with 0.7 mg/L Cd^2+^ for 10 days, the transgenic algal lines expressing MntH, HMP3, SodA, and SodC showed significantly reduced ROS accumulation compared to wild type, decreasing to approximately 69%, 70%, 57%, and 55% of the wild type, respectively (Fig. 3E), indicating that the transgenic strains had enhanced superoxide radical scavenging capacity. We further quantified the intracellular SOD and CAT activities, which showed significant increase across all strains after Cd^2+^ treatment (Fig. 3F and G). Notably, the transgenic strains demonstrated markedly higher SOD and CAT activities than those of wild type. It is hypothesized that the expression of exogenous MntH and HMP3 may promote rapid accumulation of heavy metal ions, thereby stimulating ROS generation and subsequently inducing the expression of SOD and downstream CAT. Meanwhile, the overexpression of SodA and SodC directly enhances the SOD system function and downstream CAT activity. The increase of SOD and CAT activities contributes to alleviating the intracellular oxidative stress caused by Cd^2+^ toxicity in transgenic strains, thereby benefiting cell survival and growth. In addition, we also examined the EPS production capacity of the strains under 0.7 mg/L Cd^2+^ stress. The results revealed that the stress treatment enhanced the EPS production in PCC6803, with no significant differences among strains (Fig. 3H). These findings suggested that the secreted EPS might help PCC6803 to alleviate the stress caused by Cd^2+^, while the expression of the four exogenous genes had no significant effect on the formation of EPS.

### Improved Pb^2+^ stress tolerance in transgenic PCC 6803 strains

**Fig 4:**
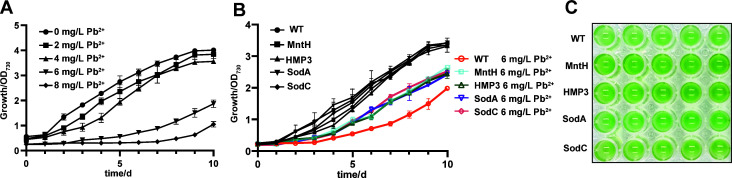
Effects of Pb^2+^ stress on growth of PCC6803 strains. (**A**) The wild-type PCC6803 was treated with varying concentrations of Pb^2+^. (**B**) Effects of 6 mg/L Pb^2+^ stress treatment on the growth of different strains. (**C**) Growth status of strains after 10 days of 6 mg/L Pb^2+^ treatment.

To investigate the effect of Pb^2+^ on *Synechocystis* sp. PCC 6803, the wild-type strain, was cultured in BG11 medium supplemented with varying concentrations of Pb^2+^, showing that Pb^2+^ affected its growth (Fig. 4A). Based on these results, a concentration of 6 mg/L was selected as the treatment concentration to study the Pb^2+^ stress tolerance of PCC 6803. As shown in Fig. 4B, the growth of the wild-type algal is severely inhibited under 6 mg/L Pb^2+^ stress, whereas the transgenic strains expressing MntH, HMP3, SodA, and SodC exhibit significant growth advantages compared with the wild type, with markedly improved growth status (Fig. 4C). The MntH transgenic strain exhibited the best resistant performance against Pb^2+^ stress.

**Fig 5:**
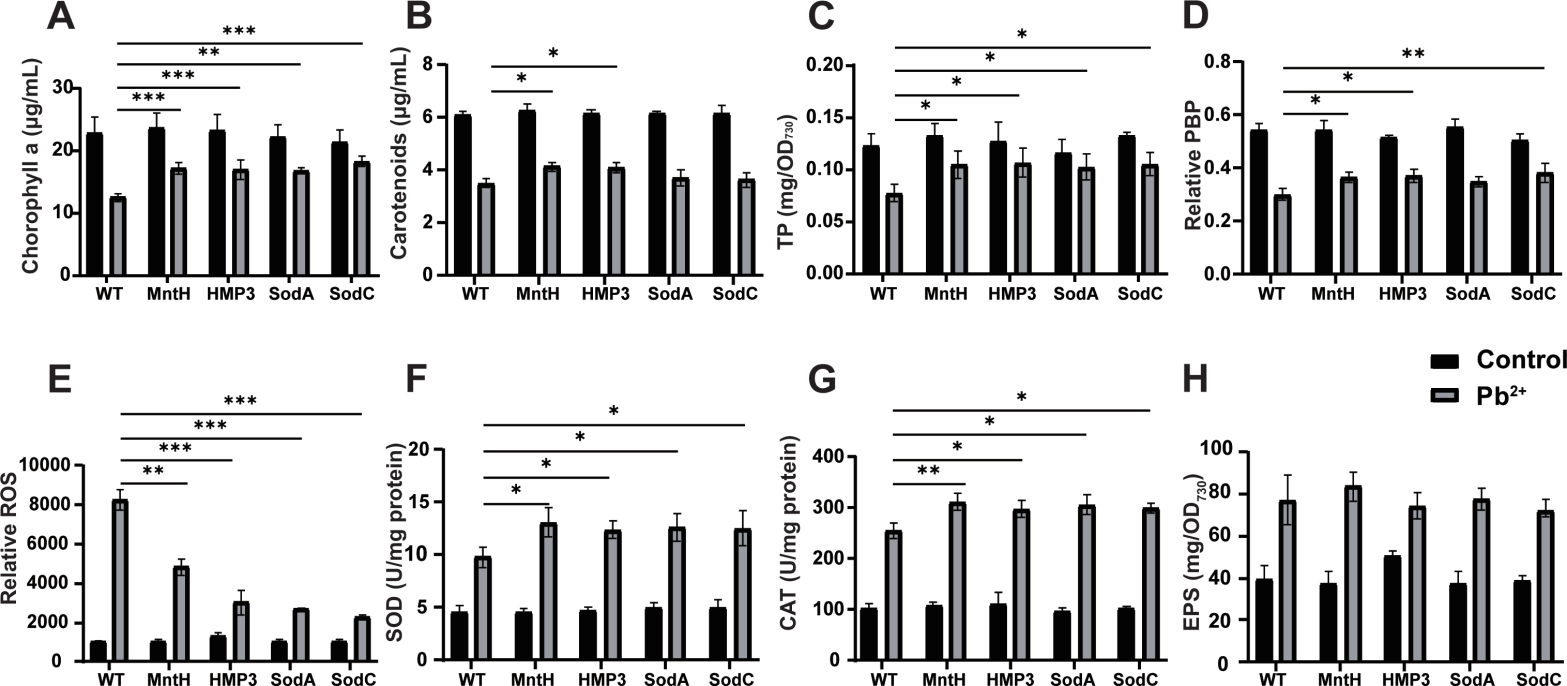
Transgenic algal strains exhibit significantly enhanced resistance to Pb^2+^ stress. After 10 days of treatment with 6 mg/L Pb^2+^, the chlorophyll (**A**), carotenoids (**B**), total protein content (**C**), relative PBP content (**D**), ROS level (**E**), SOD activity (**F**), CAT activity (**G**), and EPS content (**H**) of strains were measured. Data are shown as mean ± SEM (*n* = 3). *P < 0.05; **P < 0.01; ***P < 0.001.

To investigate the responses of transgenic PCC 6803 strains to Pb^2+^ stress, relevant intracellular physiological parameters were measured after treatment with 6 mg/L Pb^2+^ for 10 days. Under Pb^2+^ stress conditions, the damage rates of chlorophyll and carotenoids in wild-type PCC 6803 were 45.08% and 43.04%, respectively (Fig. 5A and B), while those of chlorophyll and carotenoids in the MntH, HMP3, SodA, and SodC transgenic strains were lower than those observed in the wild type. Furthermore, the total protein and PBP contents in PCC 6803 cells were also significantly impacted by Pb^2+^ stress, with damage rates reaching 36.87% and 44.70%, respectively. However, these parameters changed less in the four transgenic strains (Fig. 5C and D). The ROS level measurements revealed that the accumulation of ROS in the MntH, HMP3, SodA, and SodC transgenic strains was significantly lower than that in the wild type after exposure to Pb^2+^ (Fig. 5E). Concurrently, intracellular enzyme activity assays revealed a significant increase in SOD and CAT activities across all transgenic strains under Pb^2+^ stress (Fig. 5F and G). Notably, the SOD and CAT activities in transgenic strains were enhanced compared to those in the wild type. No significant differences were observed in the EPS composition among different strains (Fig. 5H). These results indicated that heterologous gene expression enhanced heavy metal Pb^2+^ tolerance in transgenic algae through modulation of intracellular antioxidant defense systems.

### Enhancement of Cr^6+^ stress tolerance in transgenic PCC 6803

**Fig 6:**
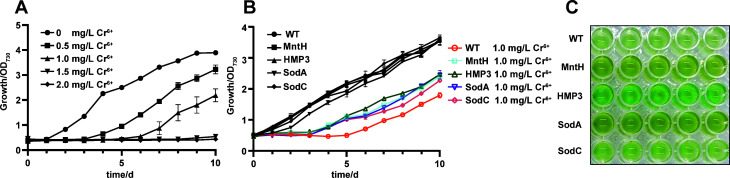
Effects of Cr^6+^ stress on growth of wild-type and transgenic PCC6803 strains. (**A**) The wild-type PCC6803 was treated with varying concentrations of Cr^6+^. (**B**) Effects of 1 mg/L Cr^6+^ stress treatment on growth of wild-type and transgenic strains. (**C**) Growth status of strains after 10 days of 1 mg/L Cr^6+^ treatment.

*Synechocystis* sp. PCC 6803 was cultured in BG11 medium supplemented with varying concentrations of Cr^6+^, and it was observed that the growth of this alga was inhibited by Cr^6+^ (Fig. 6A). A concentration of 1 mg/L was selected to investigate the tolerance of PCC 6803 to Cr^6+^ stress. As shown in Fig. 6B, under 1 mg/L Cr^6+^ stress, the growth of wild-type strain is severely inhibited with an inhibition rate of 49.22%, whereas the four transgenic strains display markedly improved growth (Fig. 6C). Among these, MntH, HMP3, and SodA exhibited almost the same inhibition rates, while SodC showed a comparatively higher inhibition rate of 35.08% (Fig. S1A).

**Fig 7:**
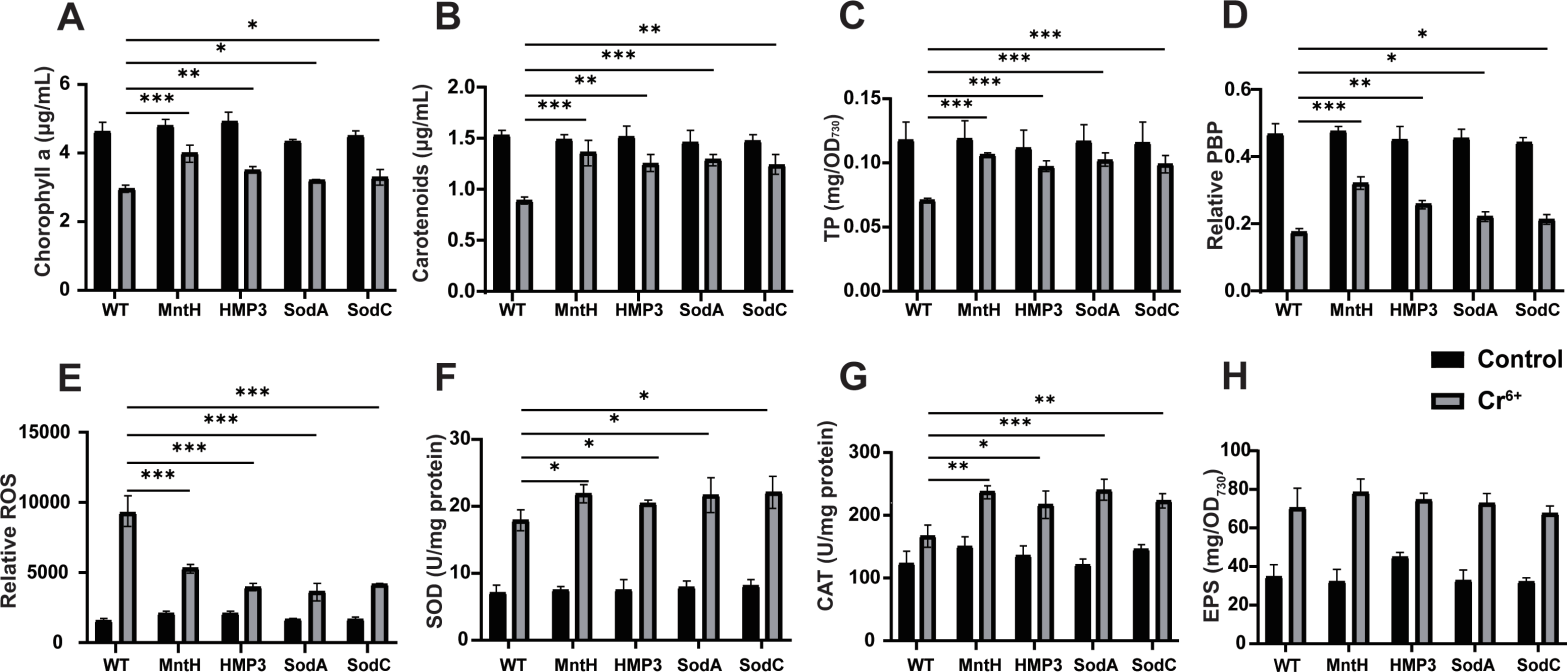
Transgenic algal strains exhibit significantly enhanced resistance to Cr^6+^ stress. After 10 days of treatment with 1 mg/L Cr^6+^, the chlorophyll (**A**), carotenoids (**B**), total protein content (**C**), relative PBP content (**D**), ROS level (**E**), SOD activity (**F**), CAT activity (**G**), and EPS content (**H**) of strains were measured. Data are shown as mean ± SEM (*n* = 3). *P < 0.05; **P < 0.01; ***P < 0.001.

To investigate the response of transgenic strains and wild-type strain to Cr^6+^ stress, relevant physiological parameters in cells were measured after treatment with 1 mg/L Cr^6+^ for 10 days. After the stress treatment, the damage rates of chlorophyll and carotenoids in wild-type PCC 6803 reached 36.22% and 41.26%, respectively (Fig. 7A and B). By contrast, MntH, HMP3, SodA, and SodC transgenic strains showed significantly lower damage rates of both chlorophyll and carotenoids compared to the wild type. In addition, intracellular total protein content and PBP content in PCC 6803 were also markedly affected by Cr^6+^ stress, and the damage rates reached 39.73% and 62.18%, respectively. However, these four transgenic strains exhibited relatively less impact on the total protein and PBP contents (Fig. 7C and D).

In the measurement of ROS levels in various algal strains after 10 days of 1 mg/L Cr^6+^ treatment, ROS accumulation was significantly lower in the MntH, HMP3, SodA, and SodC transgenic strains than that in the wild type (Fig. 7E). Further determination of intracellular SOD and CAT activities in each strain revealed that SOD and CAT activities were significantly enhanced following Cr^6+^ stress (Fig. 7F and G). Additionally, we examined the ability to form EPS in wild-type and transgenic strains under 1 mg/L Cr^6+^ stress and found no significant difference among the test strains (Fig. 7H). These results demonstrated that the expression of exogenous genes improved heavy metal Cr^6+^ tolerance of transgenic *Synechocystis* sp. PCC 6803 by modulating the intracellular antioxidant defense system.

## DISCUSSION

With the development of industrial society, the contamination of water bodies and soil by heavy metals has become increasingly severe. This contamination poses significant potential hazards to aquatic environments, potentially inducing genetic mutations in aquatic organisms, severely impacting regional aquatic ecosystems, causing substantial economic losses, and posing a major threat to human health. Consequently, the remediation of heavy metal pollution is critically important (1, 2). Bioremediation of heavy metals represents a green and sustainable remediation technology (3, 4). Among these, cyanobacteria, as highly efficient photosynthetic autotrophs with strong adaptability and adsorption capacity, serve as ideal materials for integrating photosynthetic carbon fixation with heavy metal bioremediation (4–6). However, the current bottleneck in cyanobacterial bioremediation research lies in the screening, identification, and improvement of superior strains (19). The genetic engineering of cyanobacteria to create highly robust strains represents a vital research direction. In this study, exogenous stress-resistant genes *mntH*, *HMP3*, *sodA*, and *sodC* were introduced into the genome of the model cyanobacterium *Synechocystis* sp. PCC6803 to obtain transgenic strains. The tolerance of these strains to heavy metal stress was evaluated through measurements of growth performance and physiological parameters. The results demonstrated that the transgenic strains exhibited significantly enhanced resistance to heavy metal stress, thereby preliminarily establishing the feasibility of these four stress-resistant genes as transferable functional elements for cyanobacteria and for genetically modified cyanobacterial improvement.

Under heavy metal stress, microalgal growth is inhibited, accompanied by reduced contents of photosynthetic pigments (chlorophyll a and carotenoids), which are involved in photosynthesis, as well as PBP levels that participate in photosynthetic processes. Consequently, photosynthetic activity is similarly impaired and diminished (28, 29). In this study, no significant growth difference was observed between wild-type and transgenic algal strains under normal cultivation conditions (Fig. 1G and H). However, compared to the wild type, the transgenic strains exhibited significantly improved growth performance under heavy metal stress with notably increased intracellular pigment and protein contents (Fig. 2 to 7), indicating that the expression of exogenous stress-resistant genes could help alleviate toxicity of heavy metal ions in *Synechocystis* sp. PCC6803. Cyanobacteria can adsorb heavy metal ions via extracellular polysaccharides in the environment containing heavy metal ions (30). When the concentration of heavy metal ions exceeds threshold levels, they enter cells through cation transport systems on the plasma membrane, disrupting the balance between ROS generation and scavenging and leading to an increase in intracellular ROS levels (31). Accumulated ROS stimulates the antioxidant system, causing oxidative stress and disruption of redox homeostasis balance (32). Organisms produce enzymatic and non-enzymatic antioxidants to neutralize reactive oxygen species. SOD, a metalloenzyme, converts superoxide anion radicals into hydrogen peroxide, while CAT and peroxidase subsequently oxidize hydrogen peroxide into harmless water molecules, thereby eliminating the cellular damage (33). Therefore, the ROS levels increased in all five strains under heavy metal stress (Fig. 3E, 5E and 7E). The growth rate of the wild-type strain slowed down due to the imbalance in the oxidation-antioxidation defense system. MntH protein expression facilitates Mn^2+^ transport, and Mn^2+^ can change its valence state and participate in microbial antioxidant processes as cofactors for antioxidant enzymes, such as SOD and CAT (20, 22). Consequently, the activities of SOD and CAT in MntH transgenic strains were higher than those in wild type (Fig. 3F and G, 5F and G, 7F and G). MT can bind and accumulate metal ions intracellularly (25) and scavenge free radicals to alleviate oxidative stress (34). Thus, when metal ions entered algal cells, the HMP3 transgenic strain was able to bind heavy metal ions and mitigate intracellular oxidative stress by expressing the human metallothionein HMP3 proteins. Although the activities of SOD and CAT in the HMP3 transgenic strain were slightly lower than those in MntH, SodA, and SodC (Fig. 3, 5 and 7), they still effectively enhanced the strain’s stress resistance to heavy metal ion environments. Compared to the wild type, the activities of SOD and CAT in the SodA and SodC transgenic lines increased because overexpressed SOD proteins rapidly cleared ROS to generate hydrogen peroxide, which further improved CAT activity (Fig. 3G, 5G and 7G) (22, 35), contributing to improved cellular tolerance to heavy metals. Cyanobacteria commonly absorb heavy metal by binding heavy metal cations to EPS on their cell surface (36). Although the detailed mechanism of EPS synthesis in cyanobacteria remains to be elucidated, the heavy metals are known to induce EPS production (14, 37), a phenomenon also observed in our study (Fig. 3H, 5H and 7H). However, no significant differences were detected in the EPS composition among different strains under heavy metal stress. This finding indicates that the expression of exogenous genes did not promote the EPS production in *Synechocystis* sp. PCC 6803 and that EPS was not the primary mechanism underlying the enhanced resistance in transgenic strains.

Current robustness engineering of cyanobacteria mainly improves the environmental adaptability of cells by expressing metal ion transporters, metal-binding proteins, oxidoreductases, and environmental response regulators. For instance, in 2024, Chen’s team from Tianjin University introduced phytochelatin and metallothionein into *Synechocystis* sp. PCC 6803, which improved the strain’s tolerance to multiple heavy metals and enabled effective removal of Cd^2+^, Zn^2+^, and Cu^2+^ both *in vitro* and *in vivo* (38). Their research provides a reliable reference for the engineering of cyanobacteria and their application in heavy metal bioremediation both *in vitro* and *in vivo*. In this study, the HMP3 transgenic strain constructed using similar functional elements also showed significantly enhanced tolerance to Cd^2+^, Pb^2+^, and Cr^6+^. Raghavan’s team demonstrated that overexpression of MnSOD and FeSOD in *Anabaena* sp. PCC7120 could enhance its stress resistance (39). Similarly, in this study, expression of SodA and SodC in *Synechocystis* sp. PCC6803 also significantly improved its heavy metal tolerance, with SodA exhibiting relatively stronger function. Our results indicated that three types of exogenous genes conferred resistance to heavy metal stress with varying efficiencies in *Synechocystis* sp. PCC 6803. The metallothionein HMP3 transgenic strain exhibited superior resistance to Cd^2+^ stress, as evidenced by the lowest inhibition rate (Fig. 2B and C; Fig. S1A), but demonstrated comparatively weak resistance to Pb^2+^. In contrast, the transgenic strain expressing manganese transporter MntH showed enhanced growth under Pb^2+^ stress (Fig. 4B and C). The expression of exogenous SodA conferred relatively weak resistance to Cd^2+^ and Pb^2+^ but greater resistance to Cr^6+^. Conversely, SodC conferred greater resistance to Pb^2+^ than Cr^6+^, indicating that these two SODs possess distinct functions in mitigating heavy metal stres in PCC 6803. Given that the three heavy metals exerted different levels of stress (Fig. S1A and B), a direct comparison of the efficacy of the individual genes against each stressor is challenging. Overall, the MntH transgenic strains displayed greater resistance to all three heavy metal stress compared with the other three transgenic strains. In this study, we constructed four transgenic algal strains with high stress resistance, demonstrating that MntH, HMP3, SodA, and SodC are transferable stress tolerance functional elements in cyanobacteria. This work provides material and methodological references for cyanobacterial stress tolerance engineering. However, there are limitations, such as whether these functional elements can be combined to further enhance stress resistance, which should be explored in future studies.

In conclusion, this study successfully engineered transgenic algal strains of MntH, HMP3, SodA, and SodC by integrating exogenous stress-resistant functional genes into the genome of *Synechocystis* sp. PCC6803. The experimental results demonstrated a marked enhancement in tolerance to heavy metal stress. This not only underscores the efficacy of exogenous functional genes in improving algal stress resistance but also offers both theoretical and technical foundations for the development of more robust cyanobacterial chassis cells.

## MATERIALS AND METHODS

### Culture conditions for *Synechocystis* sp. PCC 6803

The *Synechocystis* sp. PCC6803 strain was obtained from the Freshwater Algae Culture Collection at the Institute of Hydrobiology, Chinese Academy of Sciences. The strain was cultured in liquid BG11 medium supplemented with kanamycin, under continuous shaking at 200 rpm at 30℃, with a light intensity of 60 μmol photons/(m²·s) (27, 40). Solid BG11 medium was supplemented with 1.5% agar. For growth assays, cells were harvested at the stationary phase and resuspended in fresh liquid BG11 medium containing kanamycin to achieve an OD_730_ of approximately 0.3. OD_730_ was measured every 24 h using a microplate spectrophotometer (Biotek Instruments, USA) (41).

### Construction of transgenic algal strains

Using the genome of *Synechocystis* sp. PCC6803 as a template, the upstream homologous arm (up) and downstream homologous arm (down) fragments were amplified using primer pairs up-F/up-R and down-F/down-R (Table 1), respectively. The *mntH* fragment was amplified from a single colony of *Salmonella* typhimurium SL1344 using primer pair MntH-F/MntH-R. Using a single colony of *E. coli* BL21(DE3) as a template, the *sodA* and *sodC* fragments were amplified using primer pairs SodA-F/SodA-R and SodC-F/SodC-R, respectively. The human metallothionein *HMP3* gene was synthesized and used as a template to amplify the *HMP3* fragment using primer pairs HMP3-F/HMP3-R. Using the antibiotic-resistant plasmid as template, the Kan resistance cassette and promoter P_psbAII_ fragment (42, 43) were amplified using primer pairs Kan-F/Kan-R and Ppsb-F/Ppsb-R, respectively. Subsequently, these fragments were subjected to overlap PCR and cloned into the pMD19T vector via *Bgl*II and *Spe*I restriction sites. The resulting construct was transformed into *E. coli* TG1 through heat shock transformation. Colony PCR was performed using the corresponding primers to identify positive clones. The confirmed recombinant plasmids were extracted from the corresponding *E. coli* and transferred into wild-type *Synechocystis* sp. PCC6803 in log phase. After incubation at 30℃ under 60 μmol/(m²·s) light for 6–8 h, 200 µL of the incubated algal cells were evenly spread onto a mixed fibrillated ester membrane laid on non-antibiotic BG11 medium. After photobiological cultivation for 18–24 h, the membrane was transferred onto selective BG11 plates containing the corresponding antibiotic for continued photobiological cultivation. After 10–14 days, single clones were streaked onto selective BG11 plates and further cultivated. The streaked single cells were then inoculated into BG11 liquid medium containing the resistance for growth. Genomic DNA was extracted and screened by PCR using specific primers to verify the successful construction of the transgenic algal strain (41).

**TABLE 1:** Bacterial strains, plasmids, and primers used in this study^*a*^

Strains, plasmids, or primers	Genotypes, relevant characteristics, and DNA sequences	Reference or source
*E. coli*		
*E. coli* K12 MG1655	Large-scale expression recombinant plasmid	Novagen
BL21(DE3)	Template of *sodA* and *sodC*	Novagen
*S*. typhimurium	Template of *mntH*	This study
*Synechocystis* sp. PCC 6803		
*Synechocystis* sp. PCC 6803	Wild-type *Synechocystis* sp. PCC 6803	(40)
WT	*Synechocystis* sp. PCC 6803 with Km^r^ as control	This study
MntH	MntH expression in *Synechocystis* sp. PCC 6803, Km^r^	This study
HMP3	HMP3 expression in *Synechocystis* sp. PCC 6803, Km^r^	This study
SodA	SodA expression in *Synechocystis* sp. PCC 6803, Km^r^	This study
SodC	SodC expression in *Synechocystis* sp.PCC 6803, Km^r^	This study
Plasmids		
pMD19-T	Cloning vector, ColE1 replicon, Amp^r^	Takara
pMD19T-km^r^	*km^r^* in pMD19T for constructing MntH expression strain of *Synechocystis* sp. PCC 6803, Amp^r^	This study
pMD19T*-mntH*	*mntH* in pMD19T for constructing MntH expression strain of *Synechocystis* sp. PCC 6803, Amp^r^	This study
pMD19T-*HMP3*	*HMP3* in pMD19T for constructing HMP3 expression strain of *Synechocystis* sp. PCC 6803, Amp^r^	This study
pMD19T-*sodA*	*sodA* in pMD19T for constructing SodA expression strain of *Synechocystis* sp. PCC 6803, Amp^r^	This study
pMD19T-*sodC*	*sodC* in pMD19T for constructing SodC expression strain of *Synechocystis* sp. PCC 6803, Amp^r^	This study

Primer	5′−3′ sequence	Function
up-F	TTGGGTAGCAAGGAAATATCAAC	For the construction of strains
up-R	GAACGCTAAAGCCGCATTTAGGGGGCGGAATAAAAC	
Kan F	GGACTAGTAGTTGGGTAACGCCAGG	
Kan R	CACTTTATGCTTCCGGCT	
Ppsb-F	TGCGGCTTTAGCGTTC	
Ppsb-R	GGAAGATCTGGTTATAATTCCTTATGTATTTGTCGAT	
down-F	AGCCGGAAGCATAAAGTGTTGACCCACTAAAGCTCCG	
down-R	AACTCGGTCAAAATTAGTACCG	
MntH-F	GGAAGATCTATGACTGACAATCGCGTAGA	
MntH-R	GGACTAGTTTATGACAACCCCATCACCG	
HMP3-F	GGAAGATCTAGTGTTCGTTGCAACAAATTGATGA	
HMP3-R	GGACTAGTGTTCGTTGCAACAAATTGATAAG	
SodA-F	GGAAGATCTATGAGCTATACCCTGCCAT	
SodA-R	GGACTAGTTTATTTTTTCGCCGCGAAAC	
SodC-F	GGAAGATCTATGAAACGTTTTAGTCTGGCTAT	
SodC-R	GGACTAGTTTACCTTAATTACACCACAGGCATAG	
MntH-seq-F	ACGAAAATGGGATGTCGCTATTG	For qRT-PCR
MntH-seq-R	CGGTGTGGCCGCTAAAAATG	
HMP3-seq-F	GCAAATGCACCTCCTGCAAG	
HMP3-seq-R	GCTGCCTCTCCGCCTTTG	
SodA-seq-F	GACTTCGGCTCCGTTGATAACT	
SodA-seq-R	GTTTATCGCCTTTCAGCACCAGC	
SodC-seq-F	GTCTGGCTATTCTGGCGCTG	
SodC-seq-R	CAATTGACTGCCCTACCCCTTG	

^*a*^ Km^r^ and Amp^r^ represent resistance to kanamycin and ampicillin at 50μg mL^−1^.

### Quantitative real-time PCR (qRT-PCR) analysis

The *Synechocystis* sp. PCC 6803 strains were cultured in BG11 medium with 200 rpm until OD_730_ reached 1.0, and cells were collected by centrifugation (Fresco21, Thermo Scientific, USA). Total RNA was extracted with the MolPure Bacteria RNA Kit (Yeasen, Shanghai, China), and cDNA was obtained by EasyScript One-step gDNA Removal and cDNA Synthesis SuperMix (TransGen Biotech, Beijing, China), following the manufacturer’s instructions. The qRT-PCR was conducted with the SYBR Green *Pro Taq* HS Premix (Accurate Biotech, Hunan, China) with at least three replicates per group. The *rnpb* was used as an internal reference gene for the qRT-PCR analysis (41).

### Determination of growth curves under heavy metal stress

The constructed transgenic strain and the wild-type PCC 6803 were simultaneously inoculated into 10 mL of BG11 medium, followed by cultivation in a light incubator. After 10 days, the OD_730_ values were measured. After adjusting the OD values to be consistent, the cultures were uniformly transferred into 10 mL of BG11 medium supplemented with 0, 0.6, 0.8, 1.0, and 1.2 mg/L Cd^2+^. The cultures were then incubated in a light-illuminated shaking incubator with at least three biological replicates per group. OD_730_ values were measured every 24 h during continuous cultivation over 10 days to generate growth curves (44). Growth measurements under different concentrations of Pb^2+^ and Cr^6+^ ion stress were conducted using the same methodology.

### Photosynthetic pigment assay

Under heavy metal stress, the differences in photosynthetic pigments between different transgenic algal strains and wild-type strains were determined, with three replicates per group.

Two milliliters of algal culture was centrifuged at 12,000 rpm for 1 min (Fresco21, Thermo Scientific, USA) to remove the supernatant. The resulting cell pellets were resuspended in 1 mL of N,N-dimethylformamide solution (N807507-500 mL, Macklin), mixed thoroughly by pipetting, allowed to stand for 10 min, and then centrifuged again at 12,000 rpm for 1 min. The supernatant was collected, and absorbance values at 461 nm, 625 nm, and 664 nm were measured using a spectrophotometer (Biotek Instruments, USA). The total carotenoids and chlorophyll a contents were calculated according to the formula (28, 45).

Carotenoids (μg/mL) = (OD_461_ − 0.046 × OD_664_) × 4

Chlorophyll a (μg/mL) = 12.1 × OD_664_ − 0.17 × OD_625_

### Determination of ROS content

Four milliliters of algal cultures from both wild-type and transgenic strains were collected by centrifugation at 4,500 rpm for 10 min (Fresco21, Thermo Scientific, USA). The cell pellets were resuspended in PBS buffer (pH 7.4), centrifuged at 4,500 rpm for 10 min, and the supernatant was discarded. This procedure was repeated twice. Finally, the cell pellet was resuspended in 1 mL of PBS buffer. Ten micromolars of DCFH-DA were added to one aliquot of algal suspension for staining, and an equal volume of DMSO was added to another portion as control. The treated algal suspensions were incubated in a 37°C shaking incubator at 200 rpm in the dark for 30 min (45). The fluorescence intensities were detected using a microplate reader with excitation wavelength set at 488 nm and the emission wavelength at 525 nm. The OD_730_ of the algal suspension was measured using the same instrument. The normalized fluorescence intensity values were then normalized by the corresponding OD_730_ measurements to compare ROS levels across samples, with ROS content expressed as relative fluorescence intensity (12, 31).

### Measurement of extracellular polymeric substances (EPS)

Ten milliliters of algal cultures were transferred into a centrifuge tube and centrifuged at 4,500 rpm for 10 min. The supernatants were collected as soluble EPS (S-EPS). The pellet was resuspended in 1 mL of 0.05% NaCl solution, centrifuged again at 4500 rpm for 10 min, and the resulting supernatant was collected as loosely bound EPS (LB-EPS). The pellet was resuspended in 1 mL of 0.05% NaCl solution, heated in a water bath at 60°C for 30 min, and then centrifuged at 12,000 rpm for 20 min to obtain the supernatant containing tightly bound EPS (TB-EPS). LB-EPS and TB-EPS fractions were combined to obtain B-EPS. All extracted EPS fractions were pooled and filtered through a 0.45 μm membrane, and the polysaccharide content in EPS components was determined using the sulfuric acid-phenol method with glucose as the reference standard (46, 47).

### Determination of total protein content

Five milliliters of PCC6803 was harvested by centrifugation at 4°C, 8,000 rpm for 5 min. The cells were resuspended in 1 mL of PBS and disrupted using an ultrasonicator (Ultrasonic Cell Disruptor JY92-IIN, Ningbo Scientz Biotech, Ningbo, China) (power 130 W, ultrasonication 3 s, interval 3 s, total 3 min). The lysis was centrifuged at 4°C, 12,000 rpm for 10 min, followed by placing the collected supernatant on ice. Five microliters of supernatant were transferred into each well of the microplate, and 5 μL of PBS was added as a blank reference solution. One hundred microliters of Coomassie Brilliant Blue (G-250) reagent were added to both the sample and reference solutions, mixed thoroughly, and allowed to stand for 10 min. The absorbance of the samples at 595 nm was measured using a microplate reader (48).

### Determination of relative phycobiliprotein (PBP) content

Two milliliters of treated and control cyanobacteria cultures were harvested by centrifugation at 8,000 rpm for 5 min to remove supernatant. The pellet was resuspended in 25 mM HEPES (pH 7.3), adjusted to an OD_730_ of 0.5, and the OD_730_ and OD_635_ values were recorded. The adjusted suspensions were heated at 100°C in a water bath for 2 min, cooled to room temperature, and then the OD_730_ and OD_635_ were measured (33, 49, 50).

Calculate the results:

Relative PBP = (ΔOD_635_ − ΔOD_730_) / OD_(730, unheated)_

ΔOD = OD_unheated_ − OD_heated_

### Antioxidant enzyme activity assay

The supernatant from cell lysis, prepared as described in “Determination of total protein content,” above, was used to measure antioxidant enzyme activity using the Catalase (CAT) Activity Assay Kit (Sangon Biotech, Shanghai, China), according to the manufacturer’s instructions. Total SOD activity was measured using the Total Superoxide Dismutase Assay Kit with NBT (Beyotime, Shanghai, China). One unit of SOD is defined as the amount of enzyme causing a 50% inhibition of the absorbance of nitroblue tetrazolium (NBT) dye at 560 nm.

### Statistical analysis

Statistical significance was performed using one-way ANOVA with GraphPad Prism software (GraphPad Software, San Diego, California, USA). Error bars represent ± SEM. *P < 0.05; **P < 0.01; ***P < 0.001; ****P < 0.0001.

## SUPPLEMENTAL MATERIAL

Fig. S1 aem.02473-25-s0001.docx Inhibition rates of wild-type and transgenic strains under heavy metal stress.

ASM does not own the copyrights to Supplemental Material that may be linked to, or accessed through, an article. The authors have granted ASM a non-exclusive, world-wide license to publish the Supplemental Material files. Please contact the corresponding author directly for reuse.
