# Supplementary material for: Genetic engineering to improve resistance against heavy metal stress in Synechocystis sp. PCC 6803
Source: Appl Environ Microbiol. 2026 Jan 21;92(2):e02473-25. doi: 10.1128/aem.02473-25 (PMC12915332; doi:10.1128/aem.02473-25)
Supplement: Fig. S1 — Inhibition rates of wild-type and transgenic strains under heavy metal stress. [file aem.02473-25-s0001.docx]

**Genetic Engineering to Improve Resistance Against Heavy Metal Stress in *Synechocystis* sp. PCC 6803**

**Supplementary Materials**


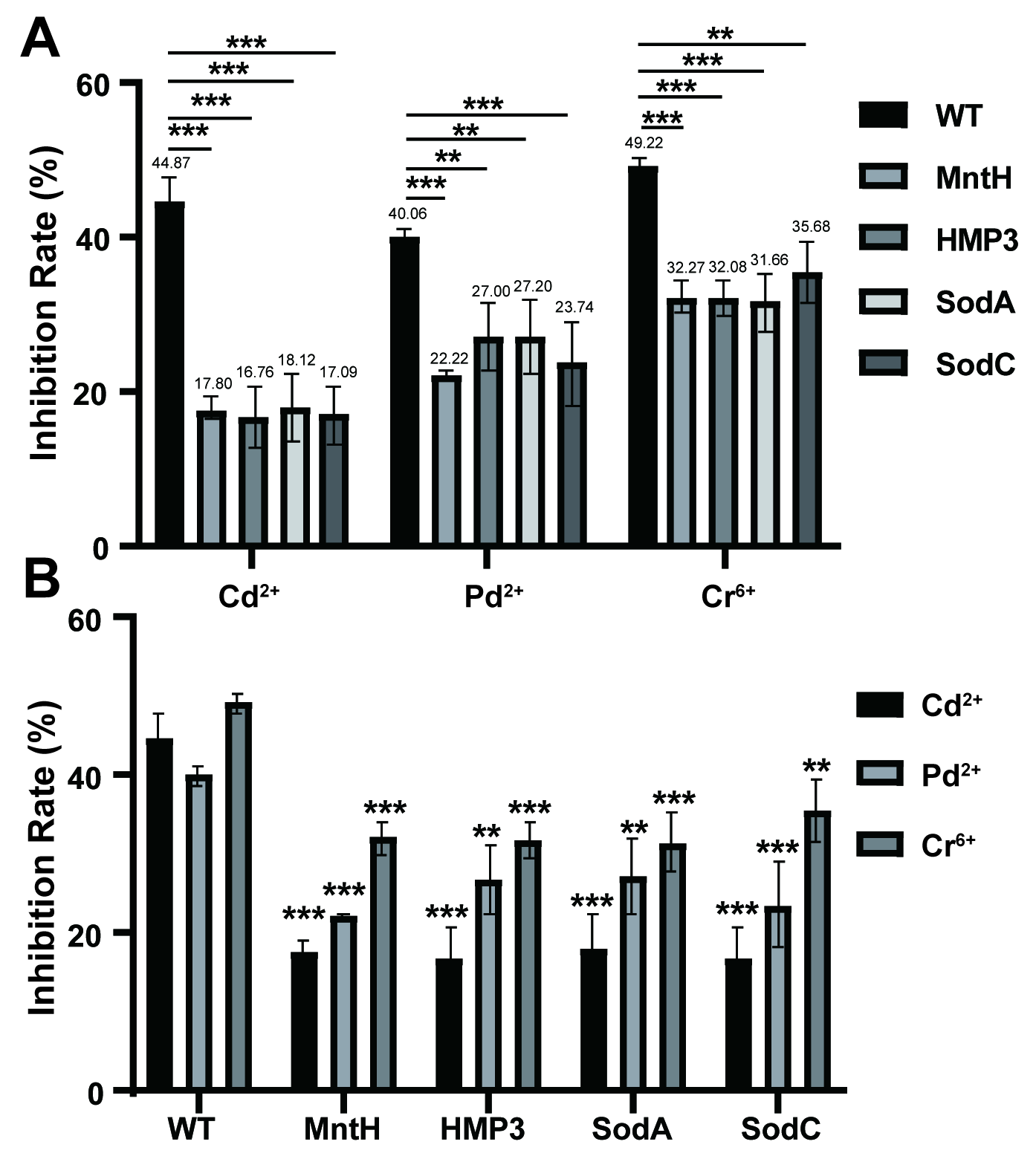


**Supplementary Figure 1. Inhibition rates of wild-type and transgenic strains under heavy metal stresses.** The inhibition rates for five strains were determined under Cd^2+^, Pd^2+^ or Cr^6+^ stress. The data are presented by heavy metals (A) or strains (B).
